# A comparison of ocular trauma scores in a pediatric population

**DOI:** 10.1186/s13104-019-4602-8

**Published:** 2019-09-11

**Authors:** Abdelhalim Awidi, Courtney L. Kraus

**Affiliations:** 1 Faculty of Medicine, University of Jordan, Queen Rania Al Abdullah Street, Amman, 11942 Jordan; 2 Pediatric Ophthalmology and Adult Strabismus Department, The Wilmer Eye Institute, The Johns Hopkins University School of Medicine, 600 N Wolfe Street, Baltimore, MD 21287 USA; 3 615 N Wolfe St, Baltimore, MD 21287 USA

**Keywords:** Ocular trauma score, Pediatric eye trauma, Eye injuries

## Abstract

**Objective:**

Pediatric ocular trauma represents a major concern for ophthalmologists. Delays in presentation, incomplete exams, inaccurate visual acuity (VA) results, and amblyopia can limit accurately predicting final visual outcomes in pediatric eye trauma. We performed a retrospective clinical study to describe the demographics and causes of eye trauma. We also compared 2 ocular trauma scoring systems, one specifically designed for pediatric trauma, to classify injuries and determine which better predicted VA outcomes. A retrospective chart review of 3 years of pediatric globe trauma was performed. Analysis was focused on mechanisms of injury and VA outcomes. Complex factors that may worsen outcomes were recorded. Ocular trauma score (OTS) and pediatric ocular trauma score (POTS) were used to assign Groups 1–5 to each case. Group 1 was poorest prognosis, Group 5 best. Association between Group and final VA was examined. Accuracy of the two systems was compared.

**Results:**

23 children met eligibility criteria (13 male). Initial VA averaged 20/200 (range no light perception (NLP)—20/20). Final VA was 20/150 (range no light perception (NLP)—20/20). Objects of injury were sharp metallic household objects (7), miscellaneous (4), toys (3), BB pellets (2), stick/wood (2), pencil/pen (1).

## Introduction

Estimates suggest that 20–50% of ocular injuries presenting to hospitals occur in children [1]. Children experiencing globe trauma can have a more complex clinical course than adults. Delay in presentation, unclear mechanisms of injury, and cooperation with eye examination can make the initial assessment less accurate. Long-term recovery can be complicated by amblyopia. Ocular trauma scores have been developed to predict outcomes and assists in triage of globe injury. The significant weight placed on initial visual acuity may inaccurately bias scores in a pediatric population. Sii et al. developed a pediatric ocular trauma score to try to offset this phenomenon [2].

## Main text

### Background

2.4 million cases of ocular trauma occur in the United States each year, of which 35% are in patients aged 17 and younger [3]. Eye injuries are a major cause of disability in all age groups, but their impact in the pediatric population is particularly heightened [4]. Common causes of pediatric ocular injuries include penetrating trauma, blunt trauma, traffic accidents, and projectile injury [5–7].

The rate of hospitalization for pediatric eye injuries in the United States in 2000 was 8.9 per 100,000 persons 20 years or younger. Males account for 69.7% of the hospitalizations [5–7]. Although most children who sustain ocular trauma do not require admission [8], those with open globe injuries have significantly poorer outcomes with more complications, surgeries, and worse overall prognosis [9–11].

While the most common causes of reduced visual acuity (VA) following trauma in children are amblyopia and corneal opacities, concerning presenting factors are numerous and include young age at presentation, poor initial VA, Zone 3 (posterior) location of injury, wound length, lens involvement, vitreous hemorrhage, retinal detachment, and endophthalmitis [12].

Various ocular trauma scoring systems have been developed to allow for prediction of final VA. Kuhn et al. developed a system using data from eye registries in the United States and Hungary [13]. This ocular trauma score (OTS) has been widely applied to numerous populations across nationalities and ages with well-validated predictive ability. Two criteria in the OTS, presenting VA and relative afferent pupillary defect (RAPD), can be challenging to obtain in children, especially those who have just sustained eye injuries. Therefore, Acar and colleagues developed a pediatric ocular trauma score (POTS) that downplayed presenting VA in its predictive model and removed RAPD [14]. The newly developed POTS included patient variables, such as age and location of injury in scoring and provided an equation to allow for scoring when no initial VA could be obtained.

The utility of a system for classification of ocular trauma is important for allowing communication between treating emergency personnel and ophthalmologists and providing information about prognosis [15–17]. Whether a separate pediatric trauma score allows for improved outcome predictions is unknown [18]. We sought to conduct a pilot investigation into which scoring system best achieves this goal using cases of pediatric eye trauma presenting to a major tertiary academic center. We used both Kuhn’s original OTS and Acar’s POTS on all cases of penetrating eye trauma and calculated which system had better prognostic accuracy.

### Methods

All cases of pediatric globe trauma presenting to the Wilmer Eye Institute at the Johns Hopkins Hospital between 2014 and 2017 were queried. The Johns Hopkins University Institutional Review Board approved this study. Patient records were reviewed to determine age, sex, complications, initial and final VA. Analysis was focused on mechanisms of injury and VA outcomes. Patients who had VA outcomes for 6 months following initial trauma were included in trauma score analysis.

OTS and POTS were calculated for each patient (Tables 1, 2). For those patients not having an initial VA, the equation 2 × (age at time of trauma + location of injury) − corresponding pathologies was used for POTS. The scores were used to assign patients a Group number between 1 and 5. Group 1 was poorest prognosis, Group 5 best. Association between Group and final VA was examined. In addition, relative predictive value of the OTS compared to POTS was assessed.

**Table 1:** Ocular trauma score (Kuhn et al.)

Group	NLP	LP-HM	1/200–19/200	20/200–20/50	> 20/40	Total
1	1	1	2	2	4	10
2		1		2	2	5
3				1		1
4						0
5						0

*NLP* no light perception, *LP* light perception, *HM* hand motion

**Table 2:** Pediatric ocular trauma score (Acar et al.)

Group	NLP	LP-HM	1/200–19/200	20/200–20/50	> 20/40	Total
1	2	2			1	5
2		1	2	3	4	10
3			2	2		4
4					2	2
5						0

*NLP* no light perception, *LP* light perception, *HM* hand motion

### Results

23 children were identified as having sustained globe trauma (13 male) and having at least 6 months of follow-up following trauma. Initial VA was able to be obtained in 16 children and averaged 20/200 (range no light perception (NLP)—20/20). Average age at time of trauma was 8.96 years old (range 5 months–16.2 years). Average age of the 7 patients not able to provide initial VA was 3.61 (range 5 months–8.2 years). Average final VA was obtained in 20 children 6 months following injury and was 20/150. The relationship between initial VA and final VA did not reach statistical significance (p = 0.07). Seven of 23 children presented to the emergency room (ER) > 48 h after initial injury. Two cases of endophthalmitis occurred, both presenting > 48 h after injury.

Objects of injury were sharp metallic household objects (7), miscellaneous (4), toys (3), BB pellets (2), stick/wood (2), pencil/pen (1). One patient presented with a retinal detachment and 2 patients suffered retinal detachments over the course of 6 months follow-up. Seven patients sustained traumatic cataracts, 5 of which required surgical removal and were left aphakic. One patient presented with a subretinal BB pellet, an afferent pupillary defect (APD), and NLP vision. The decision was made to leave the BB pellet due to poor prognosis.

Using the OTS, 16 patients could be assigned to a group. The remaining 7 patients did not have initial VA data and therefore OTS Group could not be calculated. Of the 16 patients that were able to have an OTS Group calculated, 4 did not have information on APD and therefore scoring was incomplete. There was not a strong correlation between OTS and final visual outcome (Spearman’s correlation: r = 0.005, p-value = 0.67). Table 1 shows OTS Group and final VA.

**Fig. 1:**
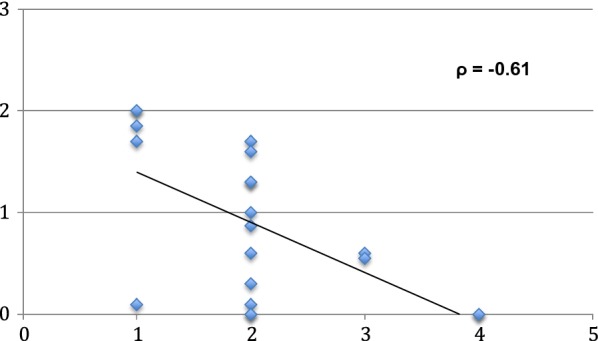
Correlation of POTS group and final VA. X-axis: final visual acuity (logmar), Y-axis: group

The POTS was calculated for all 23 patients, with the supplemental equation standing in for initial VA in 7 patients. Figure 1 shows the relationship between POTS Group and final VA. POTS correlated with visual outcomes in our population (Spearman’s correlation: r = 0.33, p-value = 0.01). Table 2 illustrates POTS Group and final VA.

### Discussion

Our population of pediatric globe trauma was similar to other reports. There was a slight male preponderance (57%) [19, 20]. Likewise, etiology of trauma was predominantly due to penetrating injury by sharp metal objects [4–10, 12, 16]. Our endophthalmitis rate was 2/23 (8.7%), not outside the scope of expectation for pediatric globe trauma [12]. Delayed follow-up (> 48 h) was seen in both of these cases; a total of 7 patients had delays in presentation.

Our objective in this review of ruptured globe outcomes was to conduct a pilot investigation into the predictive utility of the OTS to the POTS. The utility of a system for classification of ocular trauma is important for allowing communication between treating ophthalmologists and providing information about prognosis [15, 16].

Our initial investigation supports a hypothesis that the POTS may be superior to the OTS. We found the POTS to be more predictive of final visual outcomes than the OTS. We had to eliminate 7 patients from OTS calculation due to lack of initial VA data. Similarly to Schörkhuber and colleagues, when APD was not available for 4 patients we calculated the OTS without this information and an identical group score would have been given in the event all had lost points for a present APD [17]. Unlike Schörkhuber et al., we did not find that the OTS had good prognostic value in our population.

### Conclusions

Our study is limited by its retrospective nature, leading to loss of patients to follow-up and lack of outcomes data on some identified patients. There is a possibility that those not included due to limited post-globe repair follow-up differ significantly from the population available for analysis. However, the propensity to lose children to follow-up reinforces the critical need for a predictive scoring system to allow treating providers to impress upon caregivers the necessity of ongoing care. By identifying those children with more guarded prognosis early, coordinated efforts between initial treating ophthalmologist, subsequent eye care providers, parents/caregivers, social work, and school can be initiated. This can allow for targeted interventions and appropriate counseling, especially in cases of devastating monocular vision loss.

Use of a trauma score that allows for accurate prediction of final VA outcomes can be a helpful tool in the care of pediatric globe trauma. We found the POTS showed promise in its ability to more accurately predict good versus poor acuity in our pediatric population. We suggest that a large scale, multi-institutional study into the use of a pediatric ocular trauma scoring system be conducted to facilitate stratification of all childhood globe trauma.

## Limitations

- Retrospective study, leading to the possibility of losing patients to follow-up and lack of outcomes data on some identified patients.
- Possibility that those not included due to limited post-globe repair follow-up differ significantly from the population available for analysis.

## Data Availability

The datasets generated and/or analyzed during the current study are not publicly available due to institutional review board restriction but are available from the corresponding author on reasonable request.

## Abbreviations

VA: visual acuity
OTS: ocular trauma score
POTS: pediatric ocular trauma score
NLP: no light perception
ER: emergency room
